# Bilirubin levels as an independent predictor of myocarditis in patients with COVID-19

**DOI:** 10.1186/s43044-021-00234-w

**Published:** 2021-12-20

**Authors:** Muharrem Said Cosgun

**Affiliations:** Department of Cardiology, Mengucek Gazi Education and Research Hospital, Medical Faculty, Erzincan Binali Yildirim University, Erzincan, Turkey

**Keywords:** Bilirubin, Myocarditis, COVID-19, Microthrombi

## Abstract

**Background:**

Myocardial damage worsens the clinical course and prognosis of coronavirus disease 2019 (COVID-19) patients. High total bilirubin levels have been associated with a poor prognosis in COVID-19. This study aimed to investigate the predictive value of the total bilirubin level, a marker of heme oxygenase-1 enzyme activity, in determining myocarditis in patients with COVID-19.

**Results:**

A total of 190 patients diagnosed with COVID-19 were enrolled in the study. The patients were divided into two groups based on their troponin positivity. The study group (*n* = 95) consisted of patients with high troponin, and the control group (*n* = 95) consisted of patients without high troponin levels. The D-dimer (727 [572–995] vs. 591 [440–790], *p* = 0.001), C-reactive protein (CRP) (30.0 [10–48] vs. 10.3 [5.8–15.9], *p* < 0.001), and total bilirubin (9.5 [8.2–12.1] vs. 7.0 [5.3–8.0], *p* < 0.001) levels were significantly higher in the study group. In multivariate analysis, CRP (odds ratio [OR]: 1.103; 95% confidence interval [CI]: 1.060–1.148; *p* < 0.001) and total bilirubin (OR: 1.612; 95% CI: 1.330–1.954; *p* < 0.001) levels were independent predictors of myocarditis in COVID-19.

**Conclusions:**

Total bilirubin levels can be used as an early predictor of myocarditis in COVID-19 and can contribute to therapy management.

## Background

Human coronavirus (CoV) infections were considered to cause mild respiratory disease in the twentieth century [1]. However, this has changed in the first 20 years of the new century, with three outbreaks of CoVs causing significant mortality and morbidity [2]. The most important common feature of these outbreaks is that the infection becomes systemic and causes acute respiratory distress syndrome (ARDS) through autoimmune mechanisms [3, 4]. The third pandemic, called coronavirus disease 2019 (COVID-19) and caused by the severe acute respiratory syndrome (SARS)-CoV-2, started in late 2019 and continues at the time of this writing, with transmission rates higher than those of both previous CoV outbreaks [5, 6].

Existing cardiovascular comorbidities increase sensitivity to COVID-19. Moreover, COVID-19 can aggravate the underlying cardiovascular disease and lead to cardiac complications [5]. Although the infection is mild in most people, if the severe and critical form of the disease develops, the risk of multi-organ damage increases, including damage to the heart and death [7, 8]. Therefore, early diagnosis of multi-organ damage, especially heart injury, is essential. Healthcare professionals have naturally focused on lung injury, while other organ involvement, such as heart damage, has remained in the background. Troponin is the best indicator of myocardial injury due to COVID-19, as in all types of myocardial damage [9]. The available literature shows that myocardial damage worsens the clinical course and prognosis [10, 11]. For example, one study showed that more than half of the patients who died had an acute myocardial injury and that the extrapulmonary organ most commonly affected by COVID-19 was the heart [12].

Numerous reviews and meta-analyses have indicated that serum bilirubin levels are significantly high in patients with severe COVID-19 symptoms, such as pneumonia, ARDS, multi-organ damage, and septic shock [13–17]. Bilirubin is a marker of heme oxygenase-1 (HO-1) enzyme activity and is the end-product of heme reduction [18]. Free heme, the precursor of bilirubin, has been blamed in COVID-19 pathogenesis through its triggering of inflammatory processes, vascular permeabilization, and thrombosis [19]. However, total bilirubin levels are associated with both troponin increase and intracoronary thrombus burden in patients with acute coronary syndrome characterized by myocardial damage [20, 21].

### Aim

No studies in the current literature have examined the relationship between myocardial damage and total bilirubin levels in COVID-19 patients. Therefore, the purpose of this study was to investigate the relationship between total bilirubin levels, a marker of heme oxygenase-1 enzyme activity, and myocarditis in COVID-19.

## Methods

### Study population

This study was a uni-center, retrospective, descriptive, and observational study using cross-sectional data collected from the COVID-19 patients. One hundred and ninety patients whose diagnosis of COVID-19 was confirmed by reverse transcription-polymerase chain reaction (RT-PCR) were included in the study. The patients were allocated into two groups based on their troponin positivity. The consecutive patients in the study group had chest pain and elevated troponin with normal coronary angiography (*n* = 95). The control group consisted of 95 consecutive patients with similar demographic and clinical characteristics but normal troponin values. The following were exclusion criteria: (1) atherosclerotic lesion(s) in coronary angiography; (2) previous coronary artery disease; (3) referral to intensive care units due to severe and critical illness; (4) asymptomatic COVID-19 disease; (5) severe liver and kidney disease; (6) known malignancy or systemic inflammatory disease; (7) presence of anemia (hemoglobin < 13 and < 12 g/dL for men and women, respectively) or a history of blood transfusions in the last 90 days; (8) a negative RT-PCR test for COVID-19.

### Laboratory analysis

Electrocardiography measurements and troponin I levels were repeated at 4- to 6-h intervals in patients with chest pain. Serum bilirubin levels were analyzed with an autoanalyzer (AU2700 Plus analyzer, Beckman Coulter, Tokyo, Japan). Complete blood counts (hemoglobin, white blood cell, and platelet) and glucose, creatinine, creatine kinase myocardial band (CK-MB), D-dimer, and C-reactive protein (CRP) levels, and liver function tests (aspartate aminotransferase [AST] and alanine aminotransferase [ALT]) were studied in blood samples taken during admission.

### Clinical definitions

Age, gender, smoking habits, the treatment regimen for COVID-19, previous medications, and diseases were registered for both groups. The diagnoses of hypertension (HT), hyperlipidemia (HL), and diabetes mellitus (DM) were based on the previous history. The severity of COVID-19 pneumonia was defined per the guidelines of Diagnosis and Treatment of Pneumonia Caused by SARS-CoV-2- (1) mild: asymptomatic and radiologically normal patients; (2) moderate: symptomatic and patients with pneumonia on computed tomography; (3) severe: patients with fingertip oxygen saturation ≤ 93% and respiratory rate > 30/min while breathing room air; and (4) critical: patients requiring intensive care referral [22]. A level of troponin I exceeding the 99th-percentile upper reference limit was accepted as myocardial damage [11, 23].

### Statistical analysis

Categorical variables were compared with the chi-square test and shown as percentages (%). For the analysis of continuous variables, their distribution was evaluated using the One-Sample Kolmogorov–Smirnov test. The *t* test was used if the continuous variables between the two groups were normally distributed, and the Mann–Whitney *U* test was used if they were not. Continuous variables were shown as mean ± standard deviation if they were normally distributed, and as median (1st–3rd quartiles) if not. Independent predictors of myocarditis were determined by multivariate logistic regression analysis and presented with 95% confidence interval (CI) and odds ratio (OR). The cut-off value of the total bilirubin level was analyzed by the receiver-operating characteristic (ROC) curve. Statistical significance value was considered as *p* < 0.05. All data obtained were transferred to SPSS version 22 and analyzed (IBM, SPSS Statistics, USA).

## Results

**Fig. 1:**
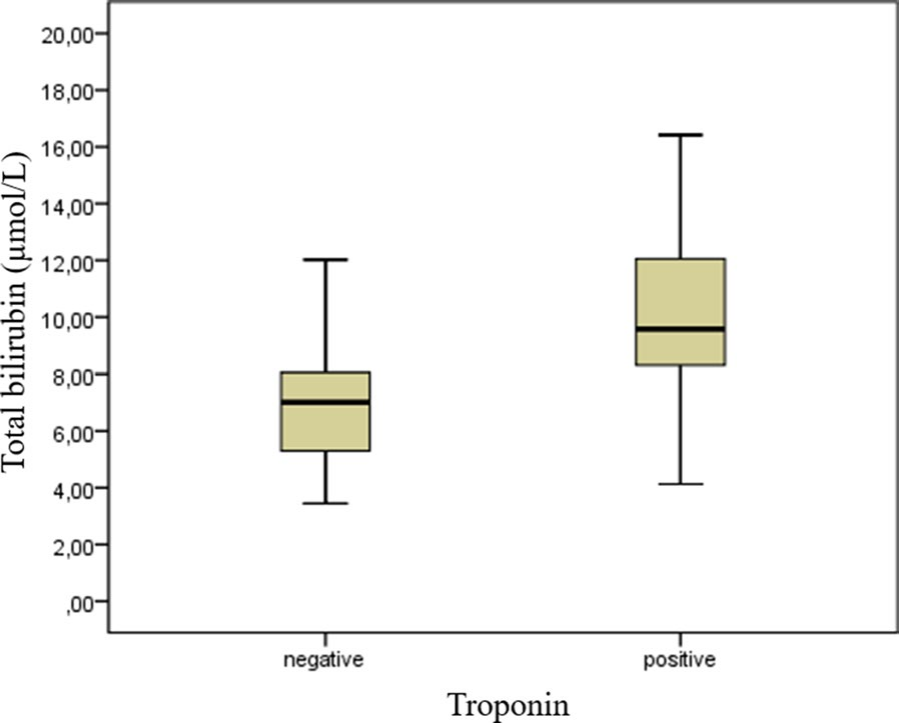
Total bilirubin levels between negative and positive troponin groups

The study group (*n* = 95; mean age 64.3 + 9.8 years; 73.7% male) consisted of troponin positive patients, while the control group (*n* = 95; mean age 62.3 + 9.3 years; 69.5% male) consisted of troponin negative patients. T able 1 shows a comparison of the baseline demographic and clinical characteristics and laboratory results. No significant difference was noted between groups in terms of age, gender, smoking habits, or previous history of HT, HL, and DM. Complete blood counts (hemoglobin, white blood cells, and platelets) and glucose, creatinine, AST, ALT, and CK-MB levels in the blood samples taken at the admission did not differ significantly between the groups. Previous medications, renin–angiotensin–aldosterone system blockers, calcium channel blockers, beta-blockers, statin, and antiaggregant were not meaningfully different between the groups. Antiviral, antibiotic, hydroxychloroquine, low molecular weight heparin, and corticosteroid treatment regimens for COVID-19 were similar between the groups. The D-dimer (727 [572–995] vs. 591 [440–790], *p* = 0.001), CRP (30.0 [10–48] vs. 10.3 [5.8–15.9], *p* < 0.001), and total bilirubin (9.5 [8.2–12.1] vs. 7.0 [5.3–8.0], *p* < 0.001) levels were meaningfully higher in the troponin-positive patients. The distribution of total bilirubin levels in the negative and positive troponin groups is shown in Fig. 1.

**Table 1:** Baseline demographic and clinical characteristics and laboratory results according to the presence of myocarditis

Variables	Troponin positive (*n* = 95)	Troponin negative (*n* = 95)	*p* value
Age, years	64.3 ± 9.8	62.3 ± 9.3	0.158
Gender, male, *n* (%)	70 (73.7)	66 (69.5)	0.52
Smoking habits, *n* (%)	22 (23.2)	20 (21.1)	0.727
Hypertension, *n* (%)	35 (36.8)	31 (32.6)	0.542
Hyperlipidemia, *n* (%)	23 (24.2)	21 (22.1)	0.731
Diabetes mellitus, *n* (%)	16 (16.8)	14 (14.7)	0.691
Hemoglobin, g/dL	13.5 ± 1.4	13.8 ± 1.1	0.122
White blood cell count, × 10^9^/L	5.9 ± 2.1	5.6 ± 2.1	0.157
Platelet count, × 10^9^/L	213 (158–235)	219 (184–262)	0.081
Glucose, mg/dL	103 (91–123)	100 (95–115)	0.465
Creatinine, mg/dL	0.82 ± 0.21	0.81 ± 0.18	0.804
Aspartate aminotransferase, U/L	30 (21–36)	29 (23–36)	0.824
Alanine aminotransferase, U/L	22 (17–29)	25 (17–41)	0.318
D-dimer, µg/L	727 (572–995)	591 (440–790)	**0.001**
C-reactive protein, mg/L	30.0 (10–48)	10.3 (5.8–15.9)	**< 0.001**
CK-MB*, U/L	19.2 (13.7–27.4)	17.7 (14.1–20.2)	0.077
Total bilirubin, µmol/L	9.5 (8.2–12.1)	7.0 (5.3–8.0)	**< 0.001**
*Previous medications*			
RAASB^†^, *n* (%)	22 (23.2)	24 (25.3)	0.735
Calcium channel blockers, *n* (%)	14 (14.7)	11 (11.6)	0.52
Beta-blockers, *n* (%)	6 (6.3)	7 (7.4)	0.774
Statin, *n* (%)	10 (10.5)	14 (14.7)	0.382
Antiaggregant, *n* (%)	9 (9.5)	11 (11.6)	0.636
*Treatment regimen*			
Antiviral, *n* (%)	86 (90.5)	83 (87.4)	0.488
Antibiotic, *n* (%)	67 (70.5)	65 (68.4)	0.753
Hydroxychloroquine, *n* (%)	25 (26.3)	22 (23.2)	0.614
LMWH^‡^, *n* (%)	89 (93.7)	87 (91.6)	0.579
Corticosteroid, *n* (%)	48 (50.5)	44 (46.3)	0.561

*Creatine kinase myocardial band

^†^Renin–angiotensin–aldosterone system blockers

^‡^Low-molecular-weight heparin

Bold defines statistically significant values

**Fig. 2:**
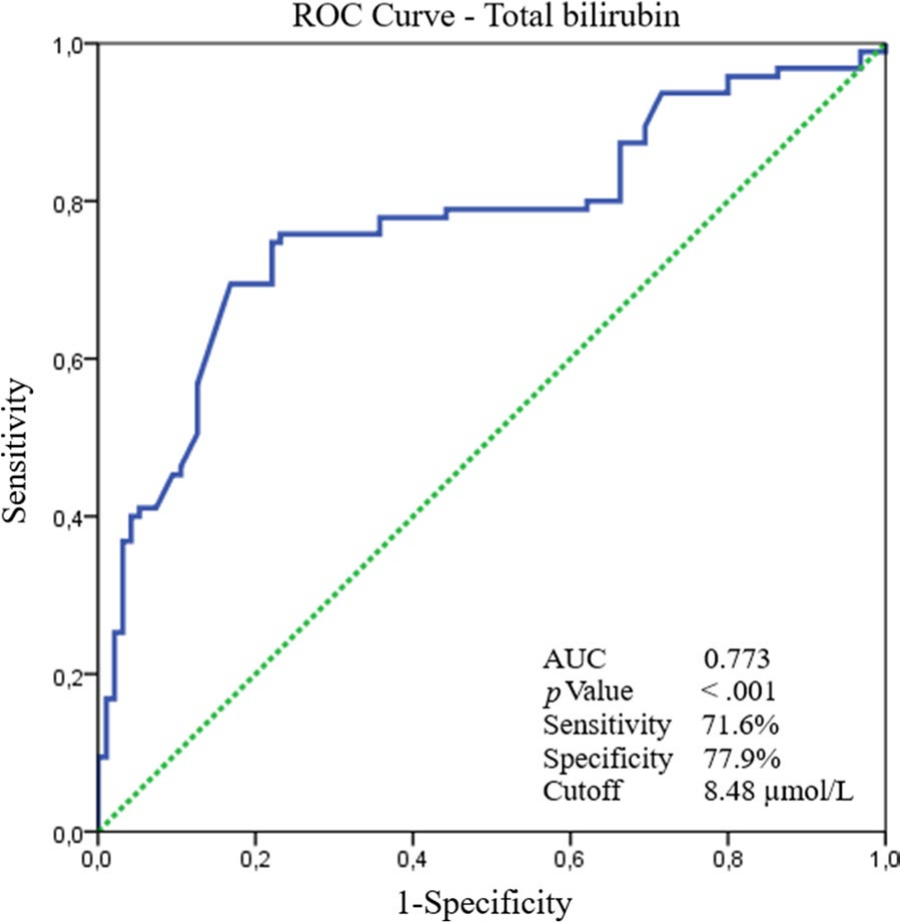
The receiver-operating characteristic (ROC) curve of total bilirubin for predicting myocarditis

Independent predictors of myocarditis were determined by multivariate logistic regression analysis. The CRP (OR: 1.103; 95% CI: 1.060–1.148; *p* < 0.001) and total bilirubin (OR: 1.612; 95% CI: 1.330–1.954; *p* < 0.001) levels were independent predictors of myocarditis in COVID-19 (Table 2). The ROC analysis revealed a cut-off value of total bilirubin for myocarditis of 8.48 µmol/L, with a sensitivity of 71.6% and a specificity of 77.9% (area under curve, 0.773; 95% CI, 0.704–0.841; *p* < 0.001; Fig. 2).

**Table 2:** Multivariate logistic regression analysis to assess predictors of myocarditis

Variables	Odds ratio (95% CI*)	*p* value
CK-MB^†^, U/L	1.002 (0.967–1.038)	0.921
C-reactive protein, mg/L	1.103 (1.060–1.148)	**< **0**.001**
D-dimer, µg/L	1.001 (1.000–1.002)	0.108
Platelet counts, × 10^9^/L	1.000 (0.995–1.005)	0.982
Total bilirubin, µmol/L	1.612 (1.330–1.954)	**< 0.001**

*Confidence interval

^†^Creatine kinase myocardial band

Bold defines statistically significant values

## Discussion

The results of our study showed that the total bilirubin levels, which are an indicator of HO-1 enzyme activity, and the CRP levels were independent predictors of myocarditis in COVID-19 patients. In addition, high D-dimer levels were correlated with myocarditis.

The SARS-CoV-2 enters host cells via endocytosis mediated by angiotensin-converting enzyme 2 (ACE-2) receptors. The virus causes ARDS due to ACE-2 receptors, commonly expressed in Type 2 pneumocytes of the lungs [24]. The same mechanism frequently targets the myocytes in the heart and enterocytes in the intestines secondary to the lungs [25]. Possible mechanisms proposed to explain the pathogenesis of myocardial injury in COVID-19 have included direct damage related to viral myocarditis, a systemic hyperinflammatory response caused by a cytokine storm, a myocardial oxygen demand–supply mismatch due to hypoxemia, down-regulation of ACE-2 receptors, systemic virus-induced endothelialitis, type 1–2 myocardial infarction, and iatrogenic effects due to corticosteroid and hydroxychloroquine use [26]. Recent autopsy-based studies have also implicated microthrombi as responsible for the etiopathogenesis of myocardial injury [27, 28]. A large-scale meta-analysis supporting this information highlighted the importance of anticoagulant therapy in COVID-19 due to the relationship between D-dimer and thrombosis [29].

Bilirubin, which has endogenous anti-oxidant and anti-inflammatory effects, is the end-product of heme catabolism. The HO enzyme catalyzes the reduction in heme groups and converts heme to carbon monoxide, ferrous iron, and biliverdin. Biliverdin is then rapidly degraded to bilirubin and excreted in the urine, while the ferrous iron is inactivated by binding to ferritin [30, 31]. Heme is highly cytotoxic due to its reactive nature, but bilirubin, the final product formed after degradation by the HO enzyme, is a scavenger of reactive oxygen species [32]. Under normal conditions, bilirubin has been shown to protect against adverse cardiovascular events due to its anti-oxidant effects [33–37]. The HO enzyme has three isoforms, with HO-1 being the rate-limiting enzyme in the heme degradation pathway. The enzyme activity of HO-1 is significantly upregulated in response to acute stress. High levels of heme and HO-1 have been detected in severe and hypoxic COVID-19 patients [38]. Based on this, HO-1 pathway immunomodulation has been viewed as a potential therapeutic strategy against COVID-19 and associated complications [39]. Conversely, the other isoforms (HO-2 and HO-3) are structural and their expression is not affected by external factors such as stress [18]. We evaluated serum bilirubin levels in the present study rather than HO-1 activity, because this analysis is inexpensive, easily accessible, and frequently used in daily practice. Okuhara et al. previously demonstrated the relationship between HO-1 enzyme activity and serum bilirubin levels in myocardial damage [40].

High serum bilirubin levels have been correlated with the burden of atherosclerosis and thrombus, low thrombolysis in myocardial infarction (TIMI) flow grade, and high in-hospital major adverse cardiac events (MACEs) in patients with ST-segment elevation myocardial infarction (STEMI) [21, 41, 42]. A correlation between total bilirubin levels and troponin has also been reported in non-STEMI patients [20]. Hamur et al. confirmed that total bilirubin levels are correlated with thrombus burden in patients with STEMI and that bilirubin is an independent predictor of high thrombus burden. Based on this, they argued that advanced anticoagulant therapy could be used in patients with elevated serum bilirubin levels and could contribute to the management of intracoronary thrombus burden [21].

As explained above, microthrombi plays an important role in COVID-19 pathogenesis and associated myocardial damage, and serum bilirubin levels are correlated with thrombus burden in stress conditions, such as acute coronary syndrome [21, 27, 28]. We designed our study based on these observations, and we determined that total bilirubin levels are an independent predictor of myocarditis in COVID-19. In addition, the CRP levels were confirmed as an independent predictor of myocarditis in COVID-19, along with bilirubin. CRP is a strong indicator of inflammation, and high serum levels are a strong and independent predictor of cardiovascular adverse events [43]. We also found significantly higher D-dimer levels in COVID-19 patients with myocarditis, consistent with previous studies. D-dimer is an indirect marker of coagulation turnover and serves as an indicator of intravascular thrombosis [44]. Previous studies have shown an association between CRP and D-dimer levels and myocardial injury in COVID-19 [45, 46].

### Study limitations

The most important limitation of this study is that we did not directly evaluate the HO-1 enzyme activity. Myocardial injury was diagnosed only by troponin elevation, and coronary angiography showed patency of epicardial coronary arteries. The absence of cardiac magnetic resonance or endomyocardial biopsy for further examination is another significant limitation. However, the pressure of the pandemic on health care may explain this situation. Another limitation is the small number of patients included in the study. It was also a retrospective study; therefore, we did not analyze long-term events.

## Conclusions

The total bilirubin level can be used as an early predictor of myocarditis in COVID-19, as it is inexpensive to measure, easily accessible, and frequently used in daily practice. Thus, it can serve as a guide for optimal anticoagulant therapy and contribute to managing high-risk patients with COVID-19. However, its potential needs to be confirmed by further clinical studies. The new point of this study is that total bilirubin level, a marker of heme oxygenase-1 enzyme activity, can predict heart damage in patients with COVID-19.

## Data Availability

The datasets used and/or analyzed during the current study are available from the corresponding author on reasonable request.

## Abbreviations

ACE-2: Angiotensin-converting enzyme 2
ALT: Alanine aminotransferase
ARDS: Acute respiratory distress syndrome
AST: Aspartate aminotransferase
CI: Confidence interval
CK-MB: Creatine kinase myocardial band
CoV: Coronavirus
COVID-19: Coronavirus disease 2019
CRP: C-reactive protein
DM: Diabetes mellitus
HL: Hyperlipidemia
HO: Heme oxygenase
HT: Hypertension
MACEs: Major adverse cardiac events
OR: Odds ratio
ROC: Receiver-operating characteristic
RT-PCR: Reverse transcription-polymerase chain reaction
SARS: Severe acute respiratory syndrome
STEMI: ST-segment elevation myocardial infarction
TIMI: Thrombolysis in myocardial infarction
